# Green Tea Extract Concurrent with an Oral Nutritional Supplement Acutely Enhances Muscle Microvascular Blood Flow without Altering Leg Glucose Uptake in Healthy Older Adults

**DOI:** 10.3390/nu13113895

**Published:** 2021-10-29

**Authors:** Ushnah S. U. Din, Tanvir S. Sian, Colleen S. Deane, Ken Smith, Amanda Gates, Jonathan N. Lund, John P. Williams, Ricardo Rueda, Suzette L. Pereira, Philip J. Atherton, Bethan E. Phillips

**Affiliations:** 1 MRC-Versus Arthritis Centre for Musculoskeletal Ageing Research and National Institute for Health Research Nottingham Biomedical Research Centre, School of Medicine, University of Nottingham, Derby DE22 3DT, UK; ushnah.din@nottingham.ac.uk (U.S.U.D.); tanvirsian@gmail.com (T.S.S.); ken.smith@nottingham.ac.uk (K.S.); amanda.gates@nottingham.ac.uk (A.G.); jon.lund@nottingham.ac.uk (J.N.L.); john.williams7@nottingham.ac.uk (J.P.W.); 2 Department of Surgery and Anaesthetics, Royal Derby Hospital, Derby DE22 3NE, UK; 3 Department of Sport and Health Sciences, College of Life and Environmental Sciences, University of Exeter, Exeter EX1 2LU, UK; c.s.deane@exeter.ac.uk; 4 Living Systems Institute, University of Exeter, Stocker Road, Exeter EX4 4QD, UK; 5 Research and Development, Abbott Nutrition, 18004 Granada, Spain; ricardo.rueda@abbott.com; 6 Research and Development, Abbott Nutrition, Columbus, OH 43219, USA; suzette.pereira@abbott.com

**Keywords:** green tea extract, epigallocatechin-3-gallate, blood flow, glucose metabolism, skeletal muscle

## Abstract

Postprandial macro- and microvascular blood flow and metabolic dysfunction manifest with advancing age, so vascular transmuting interventions are desirable. In this randomised, single-blind, placebo-controlled, crossover trial, we investigated the impact of the acute administration of green tea extract (GTE; containing ~500 mg epigallocatechin-3-gallate) versus placebo (CON), alongside an oral nutritional supplement (ONS), on muscle macro- and microvascular, cerebral macrovascular (via ultrasound) and leg glucose/insulin metabolic responses (via arterialised/venous blood samples) in twelve healthy older adults (42% male, 74 ± 1 y). GTE increased *m. vastus lateralis* microvascular blood volume (MBV) at 180 and 240 min after ONS (baseline: 1.0 vs. 180 min: 1.11 ± 0.02 vs. 240 min: 1.08 ± 0.04, both *p* < 0.005), with MBV significantly higher than CON at 180 min (*p* < 0.05). Neither the ONS nor the GTE impacted *m. tibialis anterior* perfusion (*p* > 0.05). Leg blood flow and vascular conductance increased, and vascular resistance decreased similarly in both conditions (*p* < 0.05). Small non-significant increases in brachial artery flow-mediated dilation were observed in the GTE only and middle cerebral artery blood flow did not change in response to GTE or CON (*p* > 0.05). Glucose uptake increased with the GTE only (0 min: 0.03 ± 0.01 vs. 35 min: 0.11 ± 0.02 mmol/min/leg, *p* = 0.007); however, glucose area under the curve and insulin kinetics were similar between conditions (*p* > 0.05). Acute GTE supplementation enhances MBV beyond the effects of an oral mixed meal, but this improved perfusion does not translate to increased leg muscle glucose uptake in healthy older adults.

## 1. Introduction

Increased vascular resistance [1] and endothelial dysfunction [2] are both hallmarks of advancing age and are each central risk factors for cardiometabolic disease [3]. The ensuing reductions in muscle perfusion (~20–30% in limb conduit artery blood flow compared to younger adults [4,5]) have also been posited to play a role in the aetiology of sarcopenia (i.e., age-related muscle mass and functional decline [6]), which increases the risk of frailty [7], morbidity [8] and mortality [9] and thus represents a major global health problem. Indeed, blunted muscle microvascular blood flow responses are observed in older adults following anabolic stimuli (e.g., feeding) [10,11], and nutrient-induced increases in whole-limb perfusion are attenuated with advancing age [5]. These diminished vascular responses are hypothesised to contribute to age-related “anabolic resistance” to nutrition, via the attenuated delivery and/or utility of nutrients (e.g., amino acids) and hormones (e.g., insulin) to muscle [12], although it does not fully explain anabolic resistance, since enhancing microvascular blood flow does not improve muscle anabolism [13]. Nonetheless, the ability to preserve vascular function in lifelong exercisers [14] and improve vascular function in novice exercisers [15] demonstrates that vascular dysfunction is a modifiable, rather than inevitable, aspect of chronological ageing [11]. Therefore, determining nutraceutical strategies for maintaining/potentiating vascular and metabolic responses in older adults may have significant ramifications for reducing cardiovascular disease risk and could also play a role in the maintenance of muscle health.

Epidemiological evidence has demonstrated robust correlations between green tea consumption and protection against both cardiovascular and metabolic disease [16,17,18]. The benefits of green tea are largely attributed to the naturally occurring polyphenols, particularly epigallocatechin-3-gallate (EGCg), which belongs to the catechin family and accounts for ~33–50% of the green tea solid [3,19]. At the molecular level, EGCg stimulates nitric oxide production in endothelial cells (similar to insulin [3]) primarily via the activation of endothelial nitric oxide synthase, PI3-K and Akt, resulting in nitric oxide (NO)-dependent vasodilation [3,20,21,22]. An array of pre-clinical data has directly shown that green tea/EGCg can promote vasodilation, attenuate vascular inflammation, prevent endothelial injury and ultimately enhance vascular function [22,23,24]. Compared to pre-clinical models, the corresponding effects of green tea have been less studied in humans [25]. However, from the available evidence, it has been shown that green tea-based supplements can improve endothelial dysfunction in smokers [26,27] and coronary artery disease patients [28], and can increase post-exercise blood flow in young trained men [25]. Further, the acute administration of EGCg has been shown to modulate cerebral blood flow in healthy humans [29], demonstrating the ability of green tea and/or its constituents to elicit multi-organ vascular responses.

The aforementioned green tea-induced enhancements in vascular function are the basis of the purported mechanisms underlying the subsequent improvements in insulin/glucose homeostasis, evidenced in pre-clinical models [21,30]. Should these synergistic cardiometabolic impacts of green tea be recapitulated in humans, they stand to significantly benefit health, since insulin resistance manifests with advancing age and has been shown to contribute to the development of sarcopenia [31]. Whilst there is a paucity of human data, a recent meta-analysis concluded that chronic green tea consumption may reduce fasting blood glucose levels [32], and acute supplement studies have shown green tea to improve glucose tolerance and insulin sensitivity [33], albeit not consistently [34] (and accompanying muscle perfusion measures were absent). Thus, whether green tea-induced improvements in vascular responses favourably impact insulin and glucose metabolism in healthy older adults remains to be elucidated.

Identifying efficacious nutraceuticals that impact brain, limb and skeletal muscle perfusion may improve the suboptimal glucose handling observed in ostensibly healthy older adults. Therefore, the aim of this study was to assess the impact of acute green tea extract (GTE) supplementation on oral feeding-induced changes on: (i) macrovascular (limb) blood flow, (ii) microvascular blood flow of the *m. vastus lateralis* and *m. tibialis anterior*, (iii) endothelial function, (iv) cerebral blood flow, and (v) metabolic responses in healthy older adults.

## 2. Materials and Methods

### 2.1. Ethical Approval

All the study-associated risks and procedures were thoroughly explained to the volunteers and their written consent was obtained prior to their participation. This study was reviewed and approved by the University of Nottingham Faculty of Medicine and Health Sciences Research Ethics Committee (2-1704), was conducted in accordance with the Declaration of Helsinki, and was pre-registered at clinicaltrials.gov (NCT03213340).

### 2.2. Volunteers and Study Design

Healthy older adults (≥65 years) were recruited from the local community for this randomised, single-blind, placebo-controlled, crossover trial. At the initial screening session, the volunteers’ previous medical history was discussed with a clinician, which included habitual dietary intake and known food allergies. Volunteers were considered eligible for the study if they met the following inclusion criteria: (i) ≥65 years; (ii) body mass index 18–30 kg/m^2^; (iii) free from active metabolic disease with a clinically normal blood profile (liver and kidney function; complete blood count; HbA1c < 6%); (iv) blood pressure < 160/100 mmHg; and (v) able to provide written informed consent. The exclusion criteria were: inability to adhere to the study protocol; performing regular formal exercise (or any other routine strenuous exercise) more than once a week; smoking; surgery within the past 3 months; cerebrovascular disease or active cardiovascular, respiratory, inflammatory bowel or renal disease; taking beta-adrenergic blocking agents; active malignancy or until confirmed remission; clotting dysfunction; a history of deep vein thrombosis; significant musculoskeletal or neurological disorders; a family history of early (<55 years) death from cardiovascular disease; a known sensitivity to Sonovue™ contrast agent; a known allergy or intolerance to any of the study ingredients; and/or regularly taking over-the-counter supplements containing GTE. The volunteers also completed a short physical performance battery and a handgrip strength assessment during this screening session (Table 1). Eligible volunteers were enrolled in the study and were assigned subject numbers, which were randomly assigned to either placebo (control, CON) or GTE for the first study visit. The randomisation was performed using the simple online randomisation service ‘Sealed Envelope’ (Sealed Envelope Ltd., London, UK). In line with previous work detecting supplement-induced (and non-supplement-induced) changes in microvascular responses to feeding in *n* = 10–12, a total of 12 volunteers were recruited for this study [35,36].

**Figure 1:**
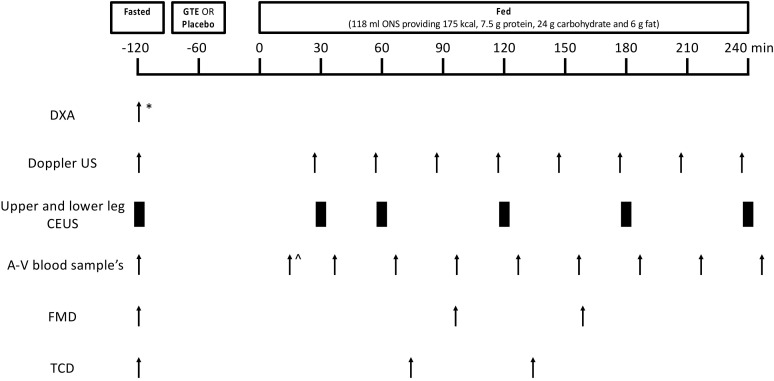
Schematic representation of the study protocol for experimental study visits 1 and 2. Twelve healthy older adults were studied in a crossover design in a fasted state, and with and without green tea extract in a fed state (via oral nutritional supplement). * indicates assessment was carried out during study visit 1 only, ^ indicates that the first blood draw occurred 15 min after the oral nutritional supplement. A-V, arterio-venous; CEUS, contrast-enhanced ultrasound; DXA, dual-energy X-ray absorptiometry; FMD, flow-mediated dilation; GTE, green tea extract; ONS, oral nutritional supplement; TCD, transcranial doppler; US, ultrasound.

For the enrolled volunteers, this study included two experimental study visits separated by a 10–15 day “wash-out” period (Figure 1). The volunteers were instructed to refrain from taking medications that may impact blood flow on the day prior to and on the day of each study visit (e.g., angiotensin-converting enzyme inhibitors, decongestants) and from heavy exercise for 48 h prior to each study visit. On the day of testing, the volunteers arrived at 0900h fasted from 2200h the night before (water ad libitum) and had their lean leg mass measured with dual X-ray absorptiometry (DXA; Luna Prodigy II; GE Medical Systems, Little Chalfont, Buckinghamshire, UK) (study visit 1 only). Baseline measures of leg blood flow (LBF) via Doppler ultrasound, microvascular blood flow (MBF) via contrast-enhanced ultrasound (CEUS), endothelial function via flow-mediated dilation (FMD), and middle cerebral artery blood flow velocity via transcranial doppler (TCD) were then performed, and a baseline blood sample was taken. All the ultrasound measures were taken using a Philips iU22 ultrasound machine (Philips Healthcare, Reigate, UK). The volunteers were then provided with the study supplement (GTE or placebo) to consume orally, with an oral nutritional supplement (ONS) consumed 60 min later. Thereafter, Doppler, CEUS, FMD and TCD measures and blood samples were obtained periodically over a 4 h period (Figure 1).

### 2.3. Study Supplements and ONS Feeding

The volunteers were randomly assigned to receive either the GTE or placebo supplement in a crossover design. The placebo condition comprised four empty capsules matched for appearance to the GTE. The GTE condition comprised four capsules, each containing GTE (300 mg) that itself contained ~45% EGCg (~135 mg) in addition to other catechins (Sunphenon, 90D, Taiyo, Japan), thus delivering a total of ~500 mg EGCg). The 500 mg EGCg dose was chosen as it is lower than doses (~800 mg EGCg) shown to be safe and very well tolerated [37], but is slightly higher than doses (~300 mg EGCg) shown to improve aspects of blood flow (e.g., FMD [28]) and glucose metabolism (e.g., fasting blood glucose [38]). It was therefore postulated to be a safe and effective acute dose. Since caffeine has been implicated in regulating vascular health [20], the GTE used was decaffeinated. In order to investigate whether GTE can enhance vascular responses beyond those achieved with mixed oral feeding, the volunteers consumed 118 mL of an ONS (Ensure Advance (Vanilla), Abbott, Zwolle, NE) providing 175 kcal (7.5 g protein, 24 g carbohydrate and 6 g fat) exactly 60 min after consuming the supplement.

### 2.4. Measurement of LBF Using Doppler Ultrasound

As previously described [39], LBF was measured by using a Doppler ultrasound (iU22 ultrasound scanner, Phillips Healthcare, Reigate, Surrey, UK). In brief, a 17–5 MHz linear probe was positioned over the left common femoral artery to facilitate the assessment of LBF as vessel cross-sectional area x mean velocity, over six cardiac cycles. To enhance the ultrasound signal, ultrasound gel was used and all the measurements were taken with the volunteer supine with no visual or aural stimuli. A mean of three measurements was made at every timepoint, dispersed across the study period. In accordance with other studies [12,40], leg vascular conductance (LVC) was calculated as: LBF/mean arterial pressure (which was calculated as: (2/3 diastolic blood pressure) + (1/3 systolic blood pressure)) and leg vascular resistance (LVR) was calculated as: mean arterial pressure/LBF.

### 2.5. Measurement of MBF Using CEUS

CEUS, as previously described in detail [41], allows the measurement of changes in MBF and its components: microvascular blood volume (MBV) and microvascular flow velocity (MFV). In brief, an iU22 ultrasound scanner (Phillips Healthcare, Reigate, Surrey, UK) was used to detect Sonovue™ microbubbles (Bracco, Milan, Italy) that were infused with an antecubital fossa vein. One L9-3 MHz linear probe was positioned on the *m. vastus lateralis* and another on the *m. tibialis anterior* to detect intravascular microbubble concentrations in both muscles. The microbubbles were disrupted using intermittent high mechanical index (MI) “flashes” with subsequent continuous low MI recordings measuring the rate of microbubble reappearance after each flash. The Sonovue™ was first infused at 2 mL/min for 1 min and then 1 mL/min for 3 min thereafter. At 2.5 min, 3× 30 s flash/replenishment recordings were performed across the last 90 s of this protocol at each CEUS timepoint. After each flash, a 0.48 s window was used to adjust for the non-contrast signal and for the rapid filling of larger conduit (non-exchange) vessels. The acoustic intensity of the insonated tissue in the post-flash period demonstrated a first order exponential association function with a rate constant that was proportional to MFV and a plateau proportional to MBV.

### 2.6. Measurement of Endothelial Function and Cerebral Blood Flow

FMD was used to assess endothelial function (right brachial artery) using standard methodology, according to the International Brachial Artery Reactivity Task Force guidelines [42]. In brief, after a baseline measurement of the brachial artery diameter for 1 min using a 17–5 MHz linear probe, arterial occlusion distal to the brachial artery was induced using a blood pressure cuff (Hokanson, Bellevue, WA, USA) inflated to 200 mmHg for 5 min. The cuff was then deflated, and the dilation of the brachial artery assessed for a further 5 min. Automated real-time arterial diameter measurements were generated through the Quipu Cardiovascular Suite FMD Studio (Quipu, Tuscany, Italy). FMD could not be recorded for two volunteers due to software failure (as such data is *n* = 10).

TCD ultrasonography was used to measure middle cerebral artery blood flow velocity as an index of cerebrovascular function [43] using standard techniques [44,45]. In brief, with all the measures performed by the same technician using a 5–1 MHz probe, the transtemporal window was located to measure middle cerebral artery (MCA) blood flow velocity (MFV). The depths of insonation were recorded so they could be duplicated during each volunteer’s second study visit, with depths between 50–60mm. It was not possible to locate the transtemporal window in four volunteers (as such data is *n* = 8).

### 2.7. Blood Glucose and Plasma Insulin

Glucose uptake/release was assessed using an arterio-venous (A-V) sampling approach. The blood glucose concentrations were measured (Glucose Analyser, YSI, Yellow Springs, OH, USA) across the leg by sampling arterialised (obtained via the “hot-hand” technique [46,47]) and venous bloods (using the Fick Principle). Plasma insulin concentrations were measured in venous blood using a high-sensitivity human insulin enzyme-linked immunosorbent assay (DRG Instruments GmbH, Marburg, Germany), according to manufacturer’s instructions. The total insulin and glucose responses to feeding for each volunteer were calculated using the area under the insulin/glucose concentration/time curve above baseline (with baseline equal to the concentration measured before supplement/feeding).

### 2.8. Statistical Analysis

Two-way repeated measures ANOVA with Sidak/Dunnett’s multiple comparison analysis was used to determine time x supplement effects. To allow comparison between conditions, the CEUS data was normalised to baseline. The area under the curve (AUC) analysis was conducted on the blood glucose A-V balance and insulin data, with paired t-tests used to determine the supplements’ effects. The data analysis was conducted using GraphPad Prism version 8 (GraphPad Software, San Diego, CA, USA), with data accepted as significant if *p* < 0.05. The data are presented as mean ± SEM.

## 3. Results

### 3.1. LBF, LVC and LVR

**Figure 2:**
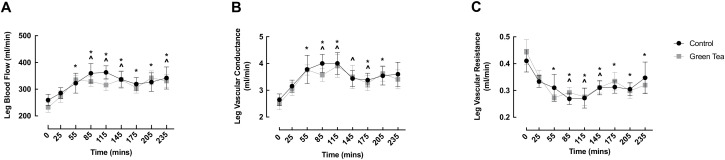
The impact of oral nutritional supplement feeding with/without green tea extract on leg blood flow (**A**), vascular conductance (**B**) and vascular resistance (**C**) in healthy older adults. ^ denotes significant difference from control baseline (*p* < 0.05); * denotes significant difference from green tea extract baseline (*p* < 0.05).

LBF significantly increased from baseline in both conditions early in the fed phase (GTE: 0 min: 232.4 ± 18.2 vs. 55 min: 335.4 ± 15.6 mL/min, *p* = 0.001; CON: 0 min: 259.6 ± 21.2 vs. 85 min: 359.4 ± 36.2 mL/min, *p* = 0.002), and was elevated at 235 min in both groups (GTE: 0 min: 232.4 ± 18.2 vs. 235 min: 331.1 ± 23.6 mL/min, *p* = 0.002; CON: 0 min: 259.6 ± 21.2 vs. 235 min: 342.1 ± 41.2 mL/min, *p* = 0.016) (Figure 2A). Interestingly, following the ONS, LBF was significantly above baseline at all timepoints in the GTE condition, but not for the CON condition, which displayed values that were no different to baseline at 175 and 205 min (Figure 2A).

LVC increased at 55 min in the GTE condition (0 min: 2.51 ± 0.23 vs. 55 min: 3.76 ± 0.19 mL/min, *p* = 0.023) and at 85 min in the CON condition (0 min: 2.65 ± 0.22 vs. 85 min: 4.00 ± 0.34 mL/min, *p* = 0.003), with both conditions returning to basal values at 235 min (GTE: 0 min: 2.51 ± 0.23 vs. 235 min: 3.41 ± 0.35 mL/min, *p* = 0.125; CON: 0 min: 2.65 ± 0.22 vs. 235 min: 3.60 ± 0.45 mL/min, *p* = 0.111).

LVR decreased in both conditions, with the onset occurring at 55 min in the GTE condition (0 min: 0.44 ± 0.05 vs. 55 min: 0.27 ± 0.01 mL/min, *p* < 0.0001), which preceded the LVR decline in the CON condition, which occurred at 85 min (0 min: 0.41 ± 0.04 vs. 85 min: 0.27 ± 0.02 mL/min, *p* = 0.001). LVR remained depressed throughout all the subsequent timepoints in the GTE condition, whereas LVR returned to baseline by 175 min in the CON condition (0 min: 0.41 ± 0.04 vs. 175 min: 0.31 ± 0.02 mL/min, *p* = 0.06) (Figure 2C). There were no significant differences between conditions at any timepoint for LBF, LVC or LVR.

### 3.2. MBV, MFV and MBF

**Figure 3:**
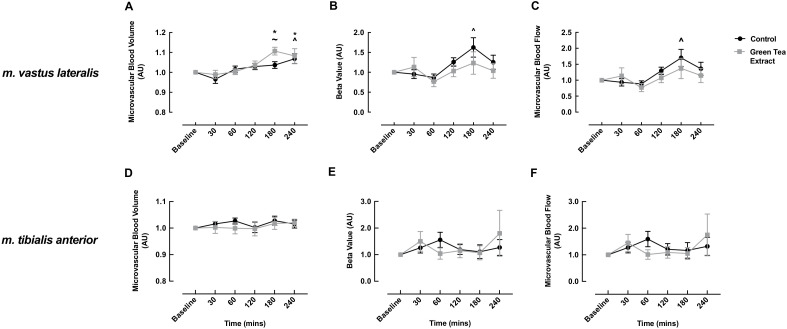
The impact of oral nutritional supplement feeding with/without green tea extract on microvascular blood volume (**A**,**D**), microvascular flow velocity (**B**,**E**) and microvascular blood flow (**C**,**F**) in the *m. vastus lateralis* (**A**–**C**) and *m. tibialis anterior* (**D**–**F**) of healthy older adults. ~ denotes a significant difference between groups (*p* < 0.05); ^ denotes significant difference from control baseline (*p* < 0.05); * denotes significant difference from green tea extract baseline (*p* < 0.05).

In the *m. vastus lateralis*, the GTE condition elicited a significant increase from baseline in MBV responses to the ONS, with this increase evident at 180 and 240 min post-meal (baseline: 1.0 vs. 180 min: 1.11 ± 0.02 vs. 240 min: 1.08 ± 0.04, *p* = 0.002). In the CON condition, MBV significantly increased from baseline at only 240 min post-meal (baseline: 1.0 vs. 240 min: 1.07 ± 0.02, *p* = 0.013). MBV was significantly higher in the GTE condition versus the CON condition at 180 min post-meal (GTE: 1.11 ± 0.02 vs. CON: 1.04 ± 0.02, *p* = 0.014) (Figure 3A).

MFV in the *m. vastus lateralis* only increased in the CON condition at 180 min (baseline: 1.0 vs 180 min: 1.62 ± 0.24, *p* = 0.003); however, there was no significant difference between the conditions at any timepoint (Figure 3B). Similarly, MBF only increased in the CON condition at 180 min post-meal (baseline: 1.0 vs 180 min: 1.70 ± 0.27, *p* = 0.001) (Figure 3C). This was largely driven by the observed increase in MFV (Figure 3B).

For the *m. tibialis anterior*, there was no significant change within or between conditions at any timepoint for MBV, MFV or MBF (Figure 3D–F).

### 3.3. FMD and TCD

**Figure 4:**
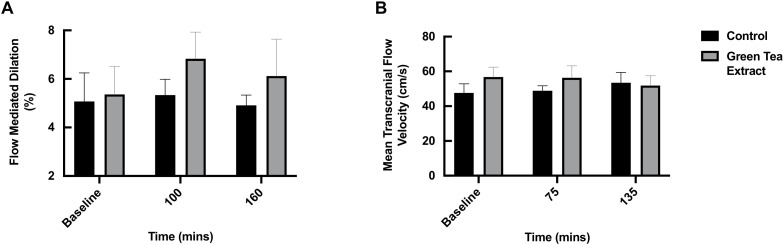
Effect of oral nutritional supplement feeding with/without green tea extract on (**A**) flow mediated dilation (*n* = 10 per condition) and (**B**) transcranial blood flow (*n* = 8 per condition) in healthy older adults.

Although there was a numerical increase in FMD in the GTE condition at 100 and 160 min following the ONS (GTE: baseline: 5.36 ± 1.15 vs. 100 min: 6.83 ± 1.10 vs. 160 min: 6.12 ± 1.52, *p* > 0.05), which was potentially indicative of improved endothelial function, the increases at neither timepoint were statistically significant, nor were there any significant differences between the groups at any timepoint (Figure 4A). There was no significant effect of the ONS with or without GTE on the TCD measurements at any timepoint, signifying no change in cerebral blood flow (Figure 4B).

### 3.4. Blood Glucose and Plasma Insulin

**Figure 5:**
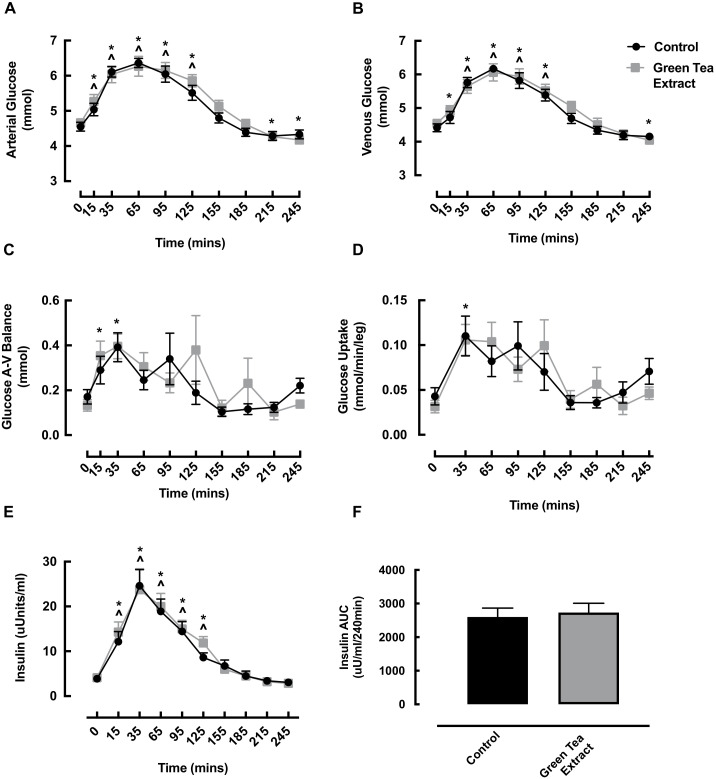
Changes in arterial glucose (**A**), venous glucose (**B**), glucose A-V balance (**C**), glucose uptake (**D**), insulin (**E**) and insulin area under the curve (**F**) in healthy older adults with/without green tea extract following oral nutritional supplement feeding. ^ denotes significant difference from control baseline (*p* < 0.05); * denotes significant difference from green tea extract baseline (*p* < 0.05). AUC, area under the curve; A-V, arterio-venous.

Arterial and venous glucose both significantly increased in the early post-feeding phase in both the GTE and CON conditions, returning to basal values by 155 min in both conditions (Figure 5A,B). Interestingly, in the GTE condition, arterial glucose was significantly reduced from baseline at 215 min and 245 min (Figure 5A) and venous glucose was significantly reduced at 245 min (Figure 5B). No significant difference was observed between the conditions at any timepoint, and arterial and venous AUC was no different between the conditions (data not shown).

Glucose A-V balance increased at 15 and 35 min in the GTE condition only (0 min: 0.13 ± 0.03 vs. 15 min: 0.35 ± 0.06 vs. 35 min: 0.40 ± 0.06 mmol, both *p* < 0.05); however, there was no significant difference between the conditions for glucose A-V balance at any timepoint (Figure 5C), or for glucose AUC (data not shown). Glucose uptake was increased in the GTE condition at 35 min only (GTE: 0 min: 0.03 ± 0.01 vs. 35 min: 0.11 ± 0.02 mmol/min/leg, *p* = 0.007); however, there was no significant difference between the conditions at any time point for glucose uptake (Figure 5D) or glucose uptake AUC (data not shown).

As expected, plasma insulin significantly increased from baseline in both conditions early post-feeding, returning to basal values by 155 min (Figure 5E). There was no significant difference between the conditions at any timepoint for insulin or insulin AUC (Figure 5E,F).

## 4. Discussion

We investigated whether acute GTE supplementation prior to oral mixed macronutrient feeding would modify vascular and subsequent metabolic responses in healthy older adults who have no known impairments in metabolic status. We found that acute GTE increased micro- but not macro-, endothelial or cerebral vascular responses to ONS; however, enhanced microvascular perfusion did not translate into improved insulin/glucose responses.

To our knowledge, this is the first study to assess the acute impact of GTE supplementation on leg muscle perfusion in healthy older adults. Our primary finding was that a high dose of GTE (containing ~500 mg of EGCg) can enhance the effect of a small mixed macronutrient meal by increasing MBV in the *m. vastus lateralis* of older healthy men and women. This demonstrates that the impact of GTE goes beyond the effects of the feeding-related insulin response, having the ability to impact vasodilation of the muscle capillaries therein increasing blood volume, and thus potentially the concentration of nutrients, in the muscle tissue. Similar to other flavonoids (e.g., cocoa flavanols [48]), it has been previously shown that green tea constituents can induce NO production in the endothelial cells, leading to vasodilation [20,21,22], which is the likely mechanism underlying the responses observed herein. Interestingly, increased MBV above that of the ONS was only observed at a late postprandial timepoint (180 min), by which time net essential amino acid uptake has normally returned to postabsorptive levels [49]. Therefore, it is likely that this response reflects late capillary recruitment and so is not expected to impact upon essential amino acid-driven increases in muscle protein synthesis, which abate ~2–3 h post-feeding [50]. Indeed, some of our previous data support this notion, whereby we found that early but not later postprandial capillary recruitment supports feeding-induced protein accretion via the delivery of EAA to the muscle [49]. As such, the question that still remains is, what are the physiological implications of enhanced ‘late’ vascular responses following acute GTE? Considering enhanced microvascular flow still represents increased nutrient, hormone and oxygen delivery to muscle (despite occurring after the “anabolic window” [50]), it is reasonable to hypothesise that such acute responses may accrue and develop into adaptations observed with chronic supplementation. For example, chronic GTE supplementation has been shown to facilitate recovery from strenuous exercise [51], improve endurance capacity via enhanced metabolic capacity and fatty acid utilisation [52] and attenuate age and diet-related muscle decline (albeit in pre-clinical models) [53,54,55], which may be related to enhanced MBV. Thus, like certain other plant flavonoids [35], we expect that GTE has important effects aside from acute protein anabolism, in the form of supporting physiological adaptations towards improving muscle health.

We also investigated MBF response in the *m. tibialis anterior*, a more oxidative and capillary-dense muscle (~70% type I fibres [56]) compared to the *m. vastus lateralis* [57], to see whether GTE is more beneficial in this muscle due to the potential for greater capillary recruitment. However, we observed no effect of GTE or the ONS on MBV in this muscle, suggesting that neither EGCg nor insulin impact the delivery of nutrients/hormones/oxygen in the *m. tibialis anterior.* However, as this is, to our knowledge, the first attempt to assess the effects of GTE in the *m. tibialis anterior*, we postulate that it is plausible that the smaller muscle size and thus vascular network of the *m. tibialis anterior* (compared to the *m. vastus lateralis*) renders potentially impactful changes smaller and harder to detect, and so our findings require validation in larger cohort clinical trials.

Despite enhancements in MBV, we observed no impact of GTE on other microvascular components, namely MFV and MBF, in the *m. vastus lateralis* or in the *m. tibialis anterior*. This, however, does not necessarily negate or lessen the efficacy of GTE for enhancing microvascular perfusion, since MBV (as opposed to other microvascular components) is confirmed to represent capillary perfusion [58,59]. It is also possible that longer-term feeding studies are needed to elucidate the effects of GTE on MFV, since MFV is dependent on the dilation of the resistance arterioles that are upstream of the terminal arterioles and capillaries. Similarly, there was no impact of GTE on macrovascular blood flow, although LBF, LVC and LVR were modulated by the ONS, suggesting that macrovascular-femoral artery flow is primarily driven by the insulin response to nutrition. Supporting our data, Ng et al. demonstrated that EGCg provision stimulated micro- but not macrovascular blood flow in rodents, leading the authors of that work to suggest that the vascular actions of EGCg (i.e., micro- vs. macrovascular) may be concentration-dependent [21]. As such, dose-response supplementation studies are needed to determine the efficacy of GTE on different aspects of human muscle blood flow.

Chronic supplementation studies have demonstrated that green tea and green tea catechins improve endothelial function in various clinical populations [27,60], with acute green tea supplementation also shown to clinically improve endothelial function [61]. Therefore, we employed FMD to investigate the impact of acute GTE (in the context of a mixed ONS) on endothelial function in our older adult cohort. Although we observed a numerical increase in FMD in the GTE condition, particularly at 100 min post-feeding, this was not significantly different, statistically, from the baseline or the control. Interestingly, Lorenz et al. found that 200 mg EGCg provided as part of a green tea beverage improved FMD in healthy men, but 200 mg EGCg provided as GTE or isolated EGCg did not [62]. The authors suggest that the caffeine within green tea that may be responsible for eliciting changes in FMD, as opposed to EGCg [20]. However, another study demonstrated the benefits of green tea (containing caffeine) for FMD but failed to demonstrate any benefit of caffeine alone [63]. The GTE used herein was purposefully decaffeinated to avoid the confounding physiological effects of caffeine, and yet a numerical increase in FMD was still observed. Considering EGCg has been shown to have dose-dependent effects on vasodilation in rodents [21], it could be that larger acute doses of GTE may significantly enhance endothelial function measured via FMD. Whilst we provided a larger dose of EGCg than is typically present in a single cup of green tea (i.e., ~500 mg vs. ~50–100 mg EGCg [64]), it is possible that more optimal GTE dosing strategies may be required to acutely enhance endothelial function. It is also possible that the chronic use of GTE is required to benefit endothelial function, since green tea containing 500 mg catechins was shown to enhance forearm blood flow compared to lower doses of catechins (80 mg) following chronic use [27]. It is also possible that our small number of volunteers precluded statistical significance, thus requiring larger clinical trials to confirm/refute the acute effects of GTE on FMD.

With regard to cerebral blood flow, we found no effect of GTE or the ONS on the TCD measurements, indicating that EGCg and/or insulin does not modulate cerebral blood flow and thus the delivery of metabolic substrates to the brain, at least in ostensibly healthy older adults. An increase in cerebral blood flow may be considered advantageous, particularly within the current population of older adults who are at a greater risk of cardiovascular and neurovascular disease, since cerebral blood flow is positively associated with cognitive performance [65]. However, among the limited available evidence to date, Wightman et al. found that an acute dose of 135 mg EGCg actually reduced cerebral blood flow when measured via near-infrared spectroscopy [29]. They postulated that the observed reduction may reflect a reduced requirement of blood flow due to improvements in other aspects of brain function by unidentified features and/or another unmeasured mechanism compensating for reduced cerebral blood flow [29]. It is clear that further clinical trials are required to clarify the current lack of consensus regarding whether GTE modulates cerebral blood flow.

Finally, we determined whether GTE-induced increases in perfusion translated into enhanced glucose metabolism/uptake, a valid area for investigation considering glucose uptake is partially mediated by microvascular responses [66] and since chronic green tea supplementation decreased fasting blood glucose levels in type 2 diabetics [38]. Despite the presence of a GTE-induced increase in MBV, we did not observe any effects of GTE on insulin/glucose kinetics. Whilst this may initially seem surprising, we assessed the acute metabolic impacts of GTE in older healthy adults, whereas pre-existing supportive evidence is largely derived from pre-clinical and/or chronic studies (e.g., [67,68]). It should also be noted that leg A-V balance reflects all the tissues within the leg, whereas we only observed an increase in *m. vastus lateralis* MBV. It is therefore possible that we did not detect metabolic changes that may have occurred in individual muscles. Nonetheless, in line with our data, others have shown that acute EGCg infusion stimulated microvascular perfusion without altering whole-body or muscle glucose uptake, albeit in rodents [21]. Interestingly, the chronic provision of green tea/EGCg increases glucose uptake and promotes GLUT4 translocation in rodents [67]. This is likely mediated via the PI3-K/Akt [69] and/or AMPK [70] pathway, as demonstrated in cultured myotubes, and so EGCg is purported to demonstrate insulin-mimetic effects (although direct evidence of insulin receptor activation is lacking [71]), at least in the chronic context. Thus, chronic supplementation studies are warranted to determine whether GTE can elicit vascular-mediated glucose handling benefits in older healthy adults.

The potential limitations of this study warrant comment. First, the exact bioavailability of the GTE is not known. The GTE was delivered as a powder in a capsule and administered 60 min prior to the ONS to account for the previously reported absorption kinetics, which demonstrated a peak in plasma catechins 60 min following the consumption of encapsulated green tea [72]. Based on this, we speculated that the administration of GTE 60 min before the ONS would allow the peak catechin levels to coincide with the insulin response to the meal, thus maximising its impact on perfusion. Furthermore, similar oral EGCg supplementation led to detectable increases in human plasma EGCg [73], demonstrating bioavailability. Second, we acknowledge that by recruiting healthy older adults only (i.e., no pre-existing disease/comorbidities), our study population may not be truly representative of the older population, particularly considering the prevalence of comorbidities and associated polypharmacy in older age [74].

## 5. Conclusions

Acute GTE supplementation significantly increases microvascular perfusion, but does not affect macrovascular perfusion, endothelial function or cerebral blood flow beyond the effects of a mixed macronutrient meal in healthy older adults. However, enhanced MBV did not impact insulin/glucose metabolism. Oral GTE is well tolerated and therefore offers a safe and efficacious nutraceutical intervention for potentiating muscle perfusion, and thus nutrient/hormone/oxygen delivery, in healthy older adults.

## Figures and Tables

**Table 1:** Volunteer characteristics (mean ± SD).

Parameter	Volunteers (*n* = 12)
Gender (% males)	42%
Age (year)	74 ± 1
Height (cm)	168 ± 13
Weight (kg)	73.4 ± 13.1
BMI (kg/m^2^)	26.1 ± 2.5
Lean mass (kg)	45.7 ± 10.5
Resting heart rate (bpm)	62 ± 6
Resting systolic blood pressure (mmHg)	136 ± 11
Resting diastolic blood pressure (mmHg)	77 ± 8
Grip strength (kg)	27 ± 8
SPPB	9 ± 2

BMI, body mass index; SPPB, short physical performance battery.

## Data Availability

The data presented in this study are available on reasonable request from the corresponding author.
